# Response to Poliovirus Outbreaks in the Lake Chad Sub-Region: A GIS Mapping Approach

**DOI:** 10.29245/2578-3009/2021/S2.1115

**Published:** 2021-04-12

**Authors:** Atagbaza Ajiri, Joseph Okeibunor, Samuel Aiyeoribe, Benoit Ntezayabo, Melinda Mailhot, Mwanza Nzioki, Alimou Traore, Abdelrahim Khalid, Mamadou Diallo, Michel Ilboudo, Bekele Mengistu Mikeyas, Dhoud Samba, Twite Mulunda, Narcisse De Medeiros, Bakoly Rabenarivo, Fabien Diomande, Sam Okiror

**Affiliations:** 1 WHO Regional Office for African (WHO AFRO), Brazzaville, Congo; 2 EHealth Africa; 3 WHO, Chad; 4 WHO, Headquarters, Geneva; 5 CDC, Atlanta; 6 Mcking Consulting Corporation; 7 UNICEF, Dakar; 8 UNICEF, Cameroon; 9 WHO Horn of Africa Coordination Office (HOA), Nairobi KENYA

**Keywords:** GIS, Coverage, Microplanning, Missed Children, Settlements, Vaccination

## Abstract

The geographic information system (GIS) mapping was used to improve the efficiency of vaccination teams. This paper documents the process in the deployment of geographical information system in response to polio eradication in Chad. It started with a careful review of government official documents as well as review of literature and online resources on Chad, which confirmed that official boundaries existed at two levels, namely Regions and Districts. All settlement locations in the target Districts were identified by manual feature extraction of high-resolution, recent satellite imagery, and map layers created for the following categories: hamlets, hamlet areas, small settlements, and built-up areas (BUAs). This clearly improved microplanning and provided valuable feedback in identifying missed settlements, leading to increased coverage and fewer missed children.

## Introduction

In 1988, the World Health Assembly launched the Global Polio Eradication Initiative (GPEI), following which WHO and partners as well as national governments in the African Region made serious commitments to reach this goal as quickly as possible^1–6^. Following these efforts polioviruses witnessed a dramatic reduction and by 2010, the initiative had limited polio endemic countries to four, including Nigeria, where known cases dropped from >1,000 in 2005 to only 21 five years later. Complete eradication of polio in Nigeria and, by implication, the Lake Chad sub region was challenging and by 2012, the number of cases climbed back to 122^7,8^. Several factors inhibited efforts at total eradication of polio in the sub region, some of the factors are high population movement, conflicts in the north eastern region of Nigeria, inaccessible or poorly visited island within the lake chad, subnational surveillance gaps in some parts of Borno state which leads to undetected circulation of poliovirus^12^. Most polio infections occurred among large nomadic populations in the northern regions of Nigeria, parts of Cameroon, Chad and Niger. A surge in violent conflict in the region since 2009 further complicated logistics^9^.

The Gates Foundation supported the GPEI to launch the geographic information systems (GIS) mapping strategies to eradicate the disease^10^. This ensured coordinate-based GIS for precise microplanning and decision making. Before now, vaccination teams relied entirely on inaccurate handdrawn maps, which led to some areas being excluded from the vaccination effort^9^. In 2010 the GIS technology was pilot tested in a project to assess the use of GIS technology to improve mapping. Its success pushed international donors to fund a larger scale initiative and GIS maps were created for all 2,238 wards in the eight northern states of Nigeria, where polio was most prevalent^7^.

By analyzing Global Positioning System (GPS) data, the central logistics team can immediately identify areas missed during immunization campaigns and can transmit this information to field vaccination teams. An online tracking system allows all stakeholders to follow vaccination coverage progress on real time basis^9^. Neighbourhoods targeted for vaccination are divided into 50m^2^ grids superimposed on a satellite image, while GPS-enabled mobile phones record the movements of vaccination teams. Green squares show visited areas and red squares missed areas (See Figure 3). GPS tracking of teams also makes data falsification less likely, since coverage is being tracked in real time^9,11^. The Nigeria experience suggests that similar projects could help eradicate infectious diseases in similarly remote, poorly mapped areas of other countries.

**Figure 1:**
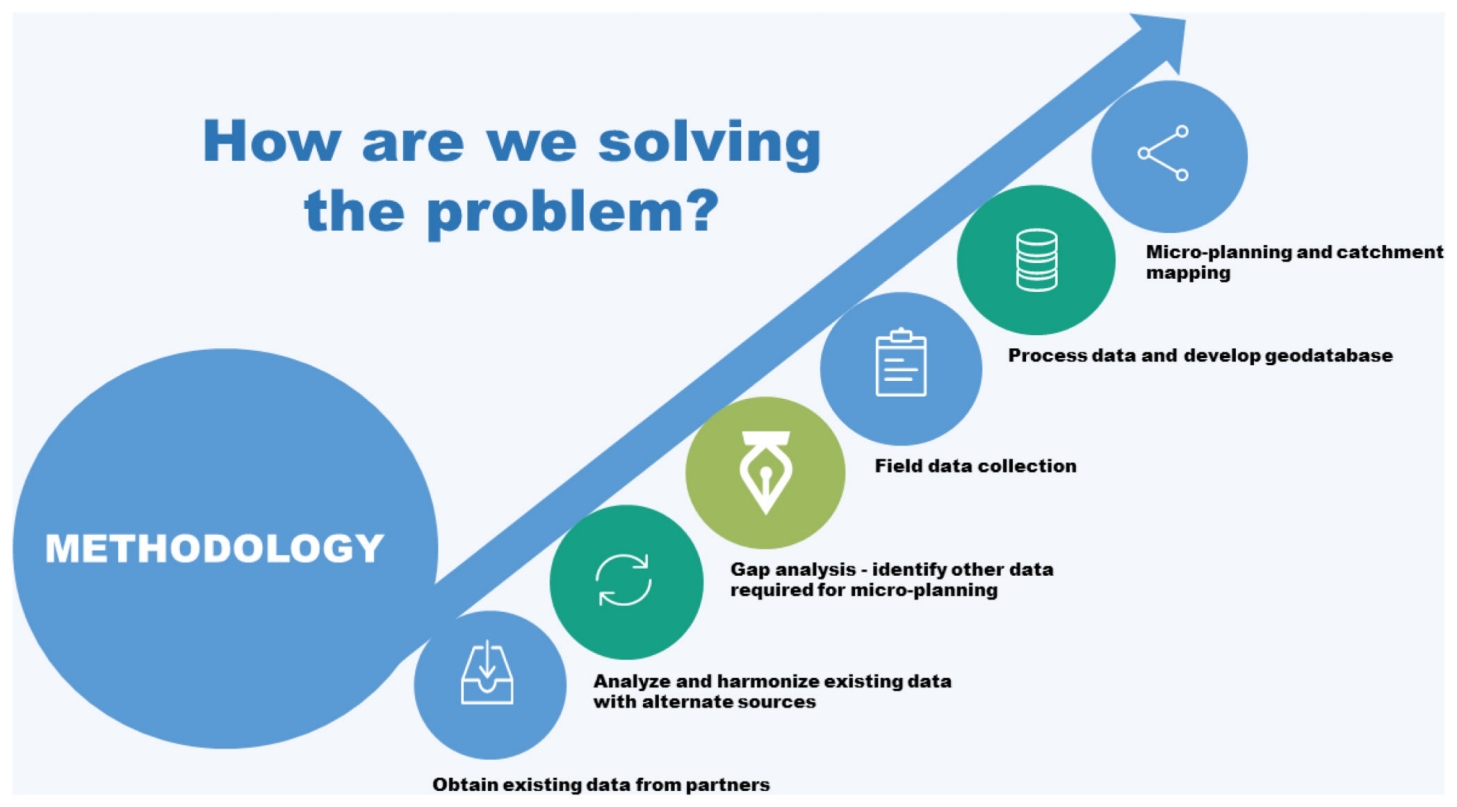
Overview of GIS Project in Chad

In Chad, the GIS mapping has been used to improve the efficiency of vaccination teams. This is very critical because slightly different from Nigeria, many of the focus settlements in Chad are in Islands, giving polio eradication efforts here a unique experience. The GIS mapping technology proved to improve the quality of microplanning, provide useful information about team performance, and provide tools to identify missed or partially covered settlements (Figure 1). This paper documents the process of use of GIS mapping since inception in Chad.

## Methods

The purpose of the Lake Chad mapping & microplanning support is to develop base maps for 31 districts within the Lake Chad Basin. This will enable the polio program within the Lake Chad Basin to improve the microplanning process by producing detailed maps and tools for effective planning and implementation of immunization activities. In Chad, two distinct groups of activities mark the GIS mapping for polio eradication, namely collecting the settlement and administrative boundary data and creating coordinate-based maps for the settlements; and integrating the maps into the microplanning process (See Figure 1). This output from these activities was later used in tracking vaccinators in the communities to identify missed settlements. The details are in reports accessible from the Lake Chad Task Team on polio eradication.

### Collecting Settlement Data, Collecting Boundary Data, and Making Maps

A careful review of government official documents as well as review of literature and online resources on Chad confirmed that official boundaries existed at two levels, namely Regions and Districts. Boundaries for the sub districts, the operational unit for polio microplanning, were not available. Worse still, the names and locations of most of the settlements, particularly in the Islands, which were high risk for polio transmission were not available for use for precise programming. All settlement locations in the target Districts were identified by manual feature extraction of high-resolution, recent satellite imagery, and map layers created for the following categories: hamlets, defined as 1–15 residences or compounds grouped together (point feature); hamlet areas, defined as groups of hamlets within 200 m of one another (polygon feature); small settlements, defined as 15–100 residences or compounds grouped together and as settlements with >100 residences or compounds that comprise huts and/or mud brick structures and lack a grid layout (point feature); and built-up areas (BUAs), defined as >100 residences or compounds with metal roofs, grouped together and organized in a grid-like, urban setting (polygon feature).

Settlement names and locations are collected by field teams, which consist of a data collector and local guide approved by the village or ward head, by using a GPS-enabled Android telephone with a custom application with the following queries: name of region (selected from the drop-down list); District name (selected from the drop-down list), settlement name (selected from the drop-down list or by entering text; collect coordinates), and points of interest (POIs; health facilities [name and type], schools [name and type], markets [name], mosques/churches [name], water access points, and the village head compound [name and telephone]; collect coordinates for each POI). This followed the approach developed in the mapping of settlement in eight Northern Nigeria States, with high risk of polio transmission^7^.

After all settlements in an Island are visited, the data are uploaded and overlaid on the satellite imagery. Any collected settlement names that do not align with an extracted feature and any extracted features that do not have a collected place name are marked for revisit/validation. A single primary name is selected for every BUA and small settlement feature. In addition, secondary names identifying subdivisions are collected for many BUAs. Naming the hamlet areas is problematic, since they are an artificial construct based on proximity. Sometimes, many names were found for a single hamlet area, and oftentimes the local guides were unable to provide names for the hamlet areas. In this case, the unnamed hamlet areas are assigned a so-called machine name (i.e., “HA-1”) until a correct local name can be identified.

Settlement area boundaries are created using an automated tool like the thiessen polygons; in ArcMap, ESRI, used to draw a border around all settlements attributed to that settlement area. To avoid litigations and disputes over properties, probably given the wrong attribution, the boundaries are referred to as “settlement area vaccination boundaries,” and it is continually emphasized that they are operational boundaries for immunization, not political boundaries. All settlement and boundary data are housed in a geodatabase, using commercially available software such as the ArcGIS Enterprise, ESRI. Finally, draft maps for microplanning are created and sent to the ward leaders for validation and sign off.

### Integrating the Maps into the Microplanning Process

**Figure 2(a-b):**
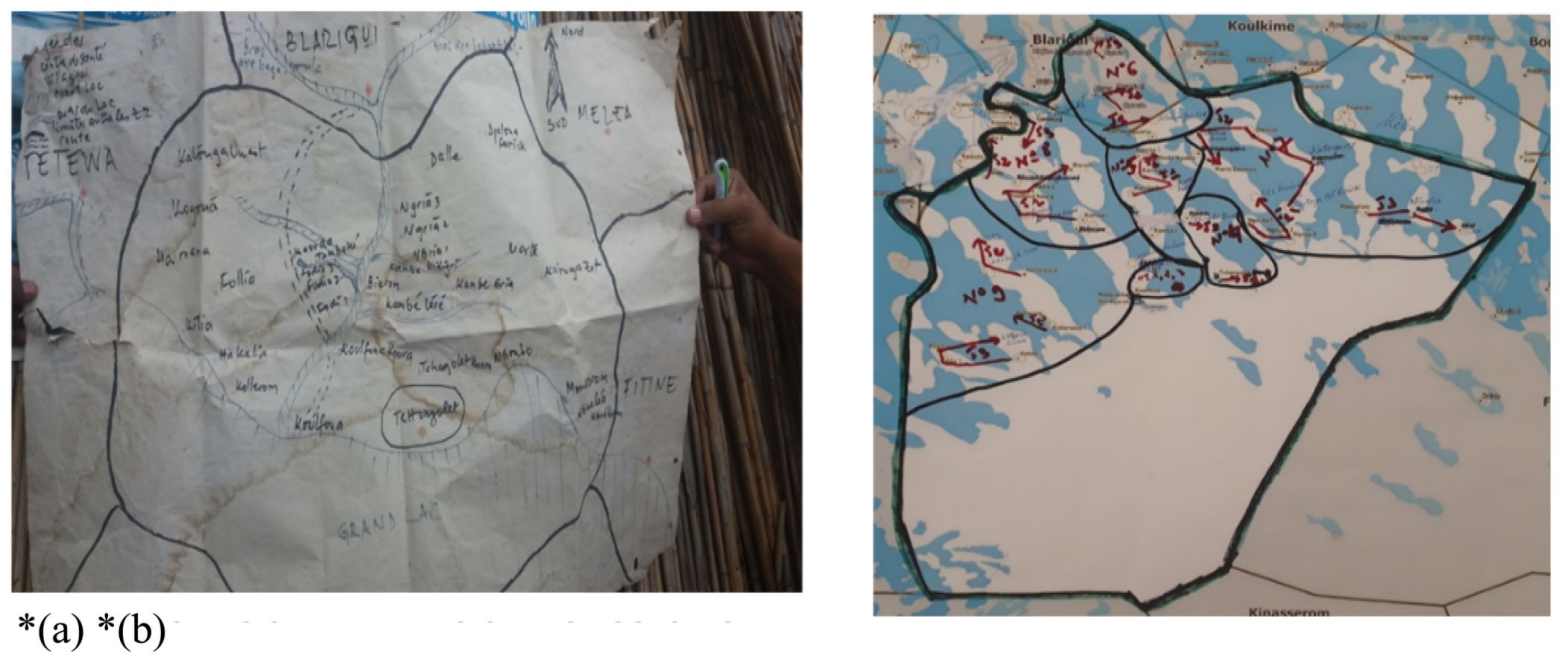
Comparison of team assignments, using hand-drawn and geographic information system maps. **(a.)** Previous hand drawn map did not highlight the logistics challenges of accessing island settlements **(b.)** New GIS based microplan map of Tchongolet health area in Bol district showing teamwork areas

Once approved by the sub district leaders following a verification exercise that involved visiting settlements, maps are printed for use in microplanning in each sub district. The maps can then be used by the sub district team, including the responsible focal point, the sub district head, and other community elders for the microplanning process. Orientation for this process is conducted through a cascaded training (See Figure 2), beginning with the regional immunization focal points and Task Team district consultants, who then train the sub district immunization officers and responsible focal points. Based on population and location, all sub district settlement areas are assigned to one or more teams on one or more days of the 4-day campaign.

### GPS Tracking, Analysis, and Feedback

GPS tracking of vaccination teams was conducted in 8 districts (approximately 49 health Areas, 1500+ island settlement and involved 491 teams) per campaign. The highest-risk districts were selected for tracking by the Task Team.

Two (2) Project Field Officers (PFO) were sent to Chad from eHealth Nigeria to support the team in Chad. They brought all necessary supplies to the field and were present during the campaign to provide training and technical support. Trained Health Center Focal Persons and subsequently trained the health district vaccination teams and oversaw deployment to the field.

The Health Center Focal Persons distributed the phones at the beginning of the campaign. Due to distance and poor network reliability, the campaign did not allow for daily collection/distribution. Instead, the vaccination team used solar chargers to ensure that the phone was always charged. Vaccinators carried the phones during the campaign and when they stopped to vaccinate for a span of two minutes, the device recorded a GPS track. (The two minutes time measurement is based on the average time a vaccinator spends vaccinating in one place. Metrics have been collected and optimized over time throughout all VTS projects to ensure accuracy of this time limit). During the campaign, the focal persons worked hand in hand with the PFO to ensure that all devices were functioning properly.

At the end of the campaign, phones were retrieved, and the information collected on the devices was synced by the PFOs. Stakeholders were able to see where each vaccination team covered through map visualization and a coverage percentage via the shared dashboard.

## Analysis

### Geographic Coverage Calculation

**Figure 3(a-c):**
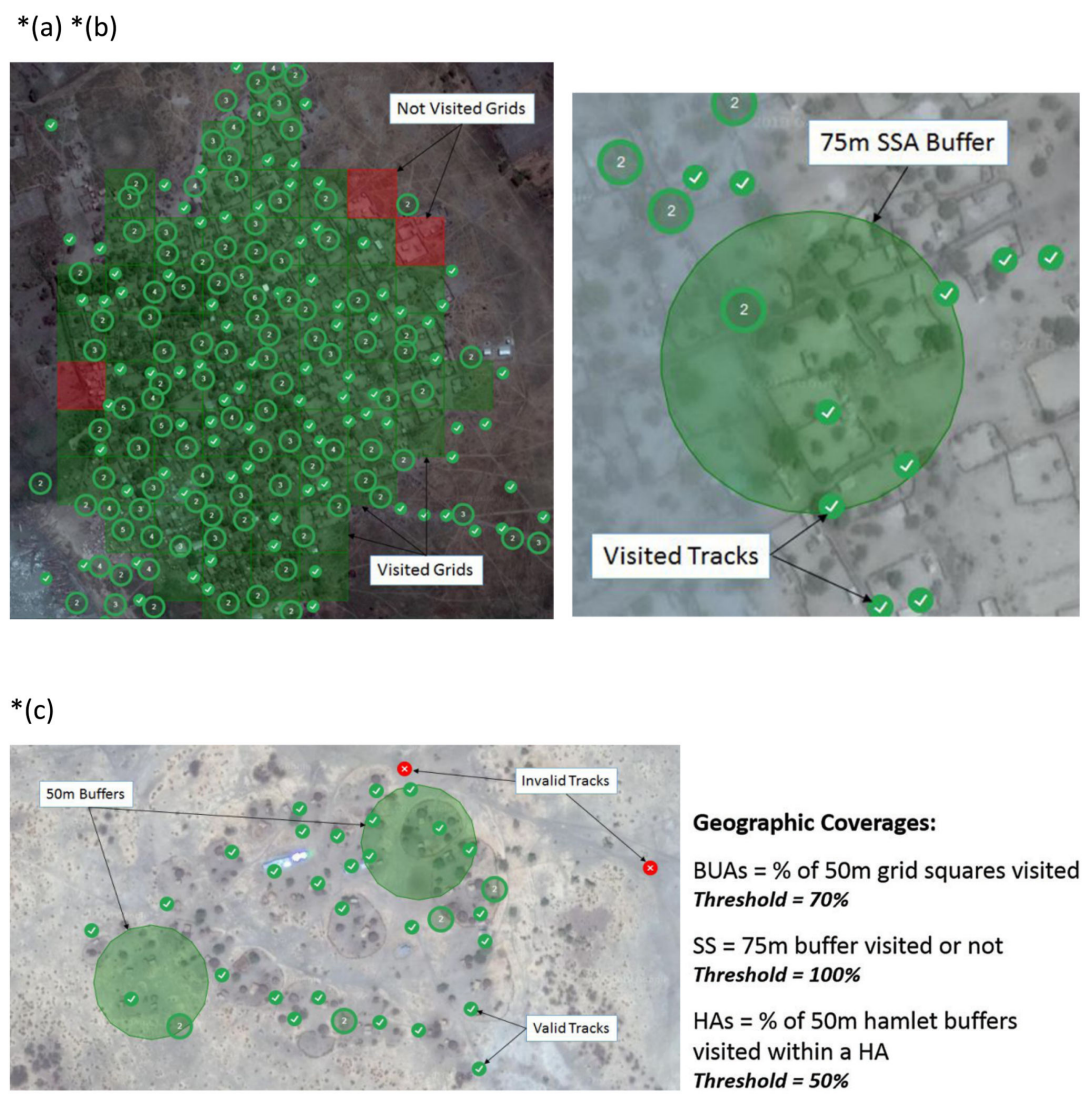
Calculation of geographic coverage for 3 settlement types (a.) *Built-up area polygon showing 96% coverage* (b.) *Small settlement area showing 100% coverage* (c.) *Hamlet area showing 100% coverage indicating that both hamlets were visited*

The settlement coverage and visitation status are ascertained when a settlement shows evidence of ‘visit’ by intersecting a valid GPS track. As part of the process for deploying the Chad VTS, GPS enabled devices are handed to the field enumerators (or vaccinators as in the case of the Polio Vaccination Tracking campaigns) to track the locations of settlements they visited. These phones have apps installed that generate passive GPS tracks every 90 seconds and these tracks are separated into valid and invalid tracks based on whether the vaccinator operated at a speed less 13 than 2m/s (valid track) or greater than 2m/s invalid track suggesting that at such speed a vaccination could not have been said to take place). Where a valid track intersects the unit for coverage calculation e.g., built-up area 50x50m grid, the settlement is said to be visited (not missed), and the percentage coverage is calculated based on the number grids visited relative to the total (See Figure 3).

The lowest unit for calculating Geographic coverage on the Chad VTS is the Settlement (referring to the Hamlet, Small settlement and Built-up areas). Settlement coverage is further aggregated at the health area, district and region administrative levels as may be required. The method for calculating settlement coverage is tied to the settlement type explained as follows:

#### Hamlet Areas’ Coverage

Hamlet areas are 200m buffers generated around Hamlet points denoting the location of hamlet settlement types. These 200m buffer polygons are dissolved where they intersect other adjoining hamlets that are within 200m and also bear the primary name of the settlement or location and in some cases a machine named settlement where no settlement name exists for that settlement.

For coverage calculation, 50m buffers are generated around the individual Hamlet settlement points within a Hamlet Area. The 50m hamlet buffers maintain a unique identifier that ties it to the respective Hamlet area it falls within, and the geographic coverage of the hamlet area is usually the percentage of visited ‘50m’ hamlet buffers within the hamlet area; calculated as total visited 50m hamlet buffer/total 50m hamlet buffers within the Hamlet area multiplied by 100(percent). Hamlet Area showing 100% coverage indicating that both hamlets were visited.

#### Built-up area Coverage

In calculating Built-up area (BUA) settlement coverage 50x50m grids representing regular sections of the BUA settlement polygon are generated within the BUA. Prior to generating the 50x50m grids, areas of the settlement representing bare ground or non-residential areas are as much as possible cropped out or excluded from the polygon area. This is to ensure that all grids are representative of actual parts of the settlement assessed. Each grid within the built-up area maintains a unique identifier (or id) that relates it with the parent built-up area.

The geographic coverage of the BUA settlement is calculated as the percentage of visited 50x50m grids within the BUA settlement area/polygon i.e., the total number of visited 50x50m grids/total 50x50m grids within the BUA multiplied by 100 (percent)

#### Small Settlement Coverage

Small settlements are identified with a point feature representing the location of the small settlements and a 75m buffer that reflects the name of the settlement and also serves as a basis for computing geographic coverage. A small settlement is said to be visited and 100% covered if it intersects a valid anywhere within the 75m buffer.

### Feedback

The dashboard data analysis identified missed, partially covered and fully covered settlements. At the end of the campaign, total settlement coverage across the eight (8) districts varied. Some of the health districts recorded significantly high coverages in ranging between of 82% and 84 % while some health districts reported the low coverage of less than 15%.

The reasons for the low coverages were documented in the lessons’ report. It should be noted though, that challenges faced while on the field from one campaign to another ranges from operational issues such as loss and broken devices to technical issues affecting settlements that could not be tracked due to security challenges. Furthermore, a core objective of the island vaccination campaign was to ensure that if one settlement is missed during one campaign, it should be covered in the subsequent campaigns, while the value of tracking system is to ensure that missed settlements can be visited before a campaign is over.

At the health area level, the coverage recorded was high with some areas having over 90%.

While the tracking dashboard does not give us the coverage of the number of children vaccinated, it provides essential and historical information tracking daily activities for each campaign day. Users are able to identify which areas were visited and understand the coverage at the settlement, health area and health district levels. This technology ensures that missed settlements are identified and geographic coverage for settlements are accurately captured against the traditional method of calculating coverage from the number of children under 5 vaccinated over the target population.

## Results

### Collecting Settlement Data, Collecting Boundary Data, and Making Maps

**Figure 4:**
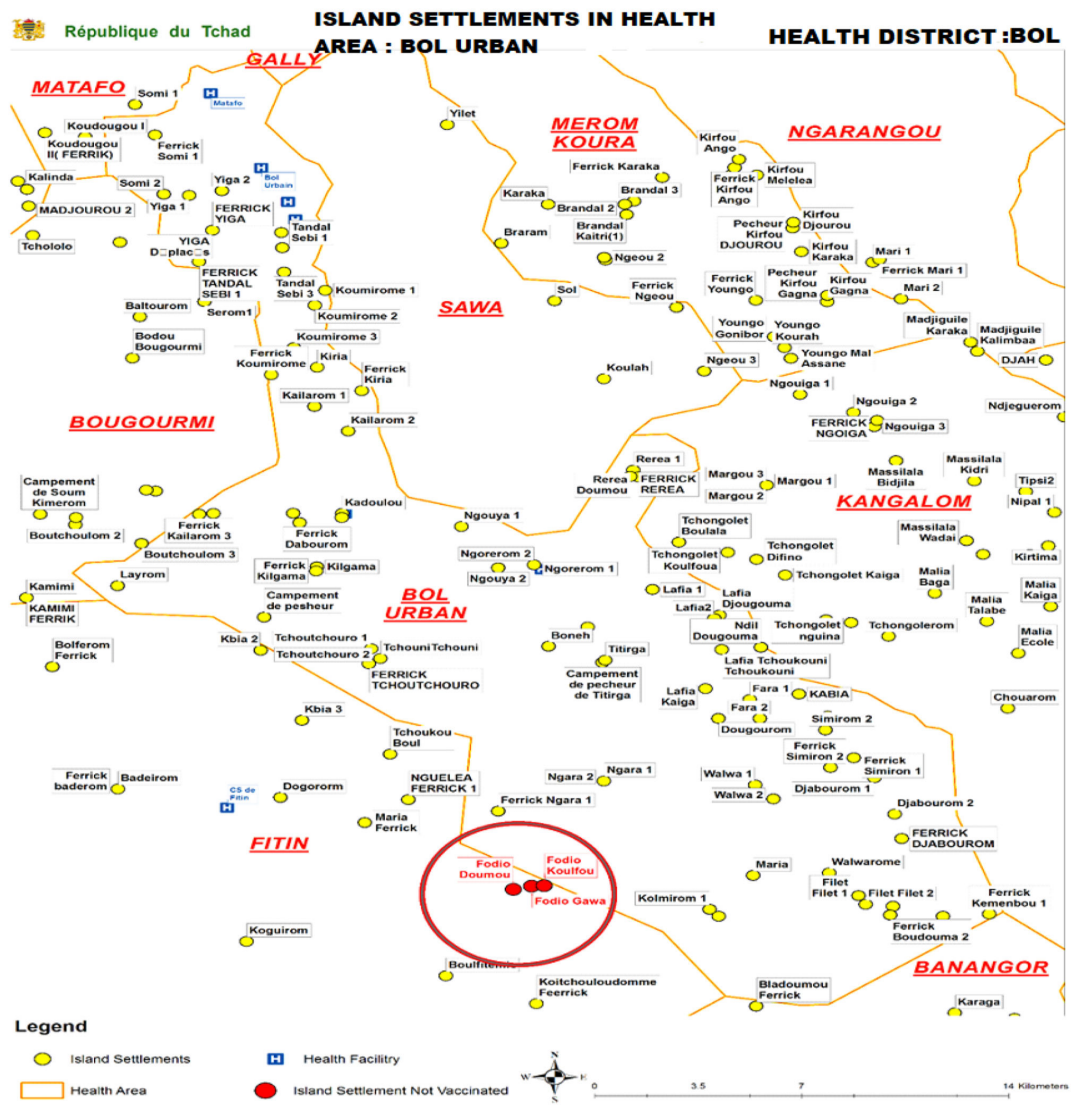
Showing location of missed settlements on the map

**Figure 5:**
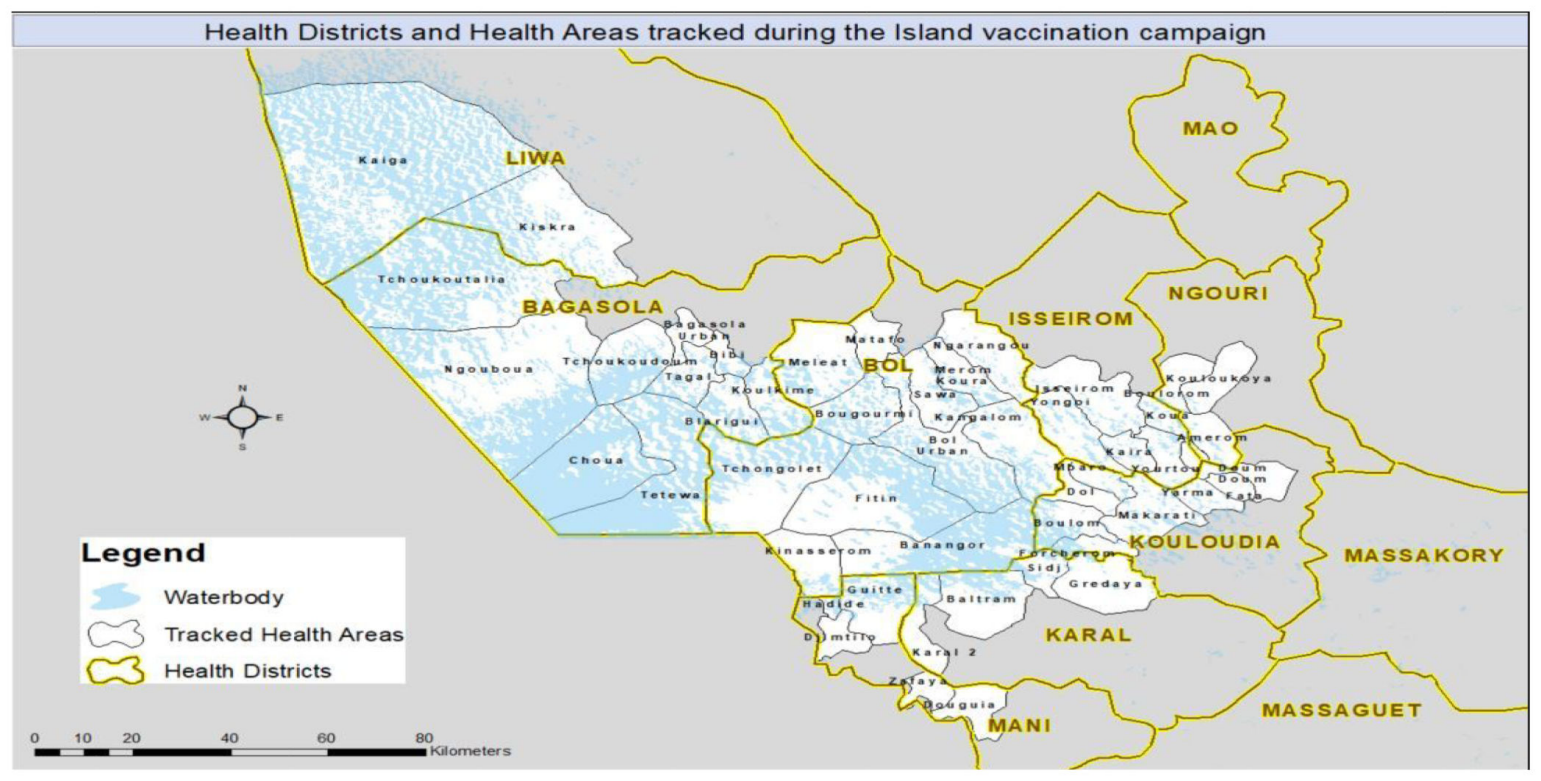
Geographic coverage of Island Vaccination areas in Chad Using GIS Technology

Given the special fact that this project was dealing with Island, there were some difficulties encountered early in the settlement data collection process. This resulted in settlement points with incorrect or missing sub district attributes, which led to misaligned health area boundaries. Many settlements were missed by vaccinators in this process (See Figures 4 and 5). The hamlet areas tend to be in hard-to-reach, remote, rural areas and were not often located in the hand drawn maps.

### Integrating the Maps into the Microplanning Process

By the end of 2018 the mapping for each settlement area was completed. Sub district maps were provided for microplanning for each campaign. At which point maps for settlements were made available for the target districts, formal orientation and training on the use of the maps took place. Sub district microplanning maps and templates were distributed to all the sub districts.

The resulting microplans included “new” settlements, hitherto missed in other maps. The GIS assisted microplans captured all settlements within the Islands of interest and allowed for more-efficient planning of team catchment areas shown in Figure 5.

## Discussion

The use of the GIS maps ensured that every settlement is included in the microplan and is assigned to a vaccination team. However, the geographic coverage does not necessarily equate to vaccination coverage. The feedback provided allows supervisors to identify these missed settlements (and poorly performing vaccination teams) and to make targeted corrective actions.

The GIS mapping in Chad has clearly improved microplanning and provided valuable feedback in identifying missed settlements, leading to increased coverage and fewer missed children. In addition, areas that have been chronically missed in the past have been identified and are now part of the microplan and can be targeted for special attention. This is similar to the outcome of using GIS maps in other projects^7^.

While the impact of this work cannot be easily separated from the many other interventions, one can conveniently attribute the successful implementation of the interventions in Island settlements in Chad to the ability of the GIS mapping’s contribution to the overall success of reaching the previously unreached settlements and reducing the proportion of zero dosed children, thus reducing the risk of polio transmission. The GIS mapping has also made it feasible to collect geographic coverage data over multiple campaigns that will permit effective assessment of the contributions of the GIS based work. Finally, the introduction of the new technology and building capacity in Chad cannot be over emphasized.
